# Assessing the Disease Activity in Crohn’s Disease, Nothing Is Enough Alone

**DOI:** 10.5152/tjg.2025.25519

**Published:** 2025-11-03

**Authors:** Ismail Inci, Zeynep Sude Celik, Haluk Tarık Kani

**Affiliations:** 1 Department of Gastroenterology, Marmara University School of Medicine, İstanbul, Türkiye; 2 Marmara University School of Medicine, İstanbul, Türkiye; 3 Institute of Gastroenterology, Marmara University, İstanbul, Türkiye

Dear Editor,

We have read the recently published article by Buldukoglu et al^1^ with great interest. Crohn’s disease (CD) is a chronic, autoimmune, and inflammatory disorder that causes inflammation and ulceration in the gastrointestinal tract.^2^^,^^3^ Patients may have diarrhea, abdominal pain, fatigue, impaired quality of life, and long-term complications such as strictures and fistulas.^3^

According to the ECCO–ESGAR (European Crohn’s and Colitis Organization - European Society of Gastrointestinal and Abdominal Radiology) joint diagnostic guideline,^4^ accurate assessment of disease activity is crucial for planning treatment and monitoring response to treatment. Endoscopy is the gold standard for mucosal assessment. Magnetic resonance enterography (MRE) (sensitivity 80%-97%, specificity 82%-95%) is used to assess transmural inflammation, while intestinal ultrasound (sensitivity 79%-94%, specificity 92%-97%) offers high accuracy and the possibility of bedside assessment. Computed tomography enterography (sensitivity ~81%, specificity ~88%) is similar but is limited due to radiation exposure. Small bowel capsule endoscopy is as accurate as MRE/ultrasound and is particularly effective in detecting proximal disease, but it is retained in 1.5%-2% of cases. Fecal calprotectin (FC) correlates with endoscopic activity (sensitivity ~78%, specificity ~73% for predicting recurrence), while C-reactive protein (CRP) is a rapid but less sensitive marker.

Scores most commonly used to assess clinical activity in patients with CD are the Crohn’s Disease Activity Index (CDAI) and the Harvey–Bradshaw Index (HBI).^5^ Although CDAI and HBI are useful for monitoring luminal inflammatory activity, they may underestimate clinical activity in cases of perianal and fistulizing CD. For example, in the original definition of CDAI, the presence of an actively draining fistula contributes minimally, while HBI does not directly score perianal findings. In colonic involvement, the symptom profile (e.g., diarrhea, abdominal pain) is generally reflected in the scores, so performance is relatively better; however, it is not possible to reliably distinguish the degree of inflammation with CDAI/HBI alone, and therefore, it is recommended that they be supported by objective biomarkers or imaging methods.^5^

There is no single or specific clinical or biochemical parameter that is sufficient to reflect disease activity. This has led to a search for new parameters.^6^ Based on this gap, the authors present Gasdermin D (GSDMD) as an inflammatory biomarker that can be used to predict disease activity in CD within the framework of their hypothesis. However, the authors evaluated the disease activity by HBI. Colonoscopic evaluation and scoring are crucial to assess disease activity in CD. Also, in fistulizing disease, sometimes additional imaging modalities and scores are needed to assess the disease activity more accurately.^7^ Correlation of GSDMD with HBI is important to show its function in assessing disease activity, but it is not enough. Colonoscopic evaluation would be better to assess its correlation with disease activity, even though the authors stated this as a limitation. Besides this, in patients with perianal involvement, imaging scores such as van Assche Index or MAGNIFI-CD are needed to assess the activity, and it is important to evaluate the correlation of GDSMD.^8^^,^^9^

In conclusion, there are indices such as CDAI and HBI, but they are not sufficient. There is a need for applicable, sustainable, and inclusive indices. Also, there are biomarkers such as CRP and FC, but again, they are not sufficient alone. All parameters used to assess disease activity must correlate with endoscopic data. Research into potential new non-invasive indices and biomarkers is valuable for routine use and should be encouraged because, although endoscopy is a powerful tool, it is invasive.

## Data Availability

The data that support the findings of this study are available on request from the corresponding author.
